# Oligomer Formation of Tau Protein Hyperphosphorylated in Cells*

**DOI:** 10.1074/jbc.M114.611368

**Published:** 2014-10-22

**Authors:** Katharina Tepper, Jacek Biernat, Satish Kumar, Susanne Wegmann, Thomas Timm, Sabrina Hübschmann, Lars Redecke, Eva-Maria Mandelkow, Daniel J. Müller, Eckhard Mandelkow

**Affiliations:** From the ‡ DZNE, German Center for Neurodegenerative Diseases, 53175 Bonn, Germany,; the § CAESAR Research Center, 53175 Bonn, Germany,; the ¶ Department of Biosystems Science and Engineering, ETHZ, 4058 Basel, Switzerland,; the ‖ Institute of Biochemistry, Faculty of Medicine, Justus-Liebig-University, 35390 Giessen, Germany, and; the ** Joint Laboratory for Structural Biology of Infection and Inflammation, University of Hamburg and University of Lübeck, ℅DESY, 22603 Hamburg, Germany

**Keywords:** Fluorescence Anisotropy, Neuron, Phosphorylation, Synapse, Tau Protein (Tau), Oligomers, Time-correlated Single Photon Counting, Toxicity

## Abstract

Abnormal phosphorylation (“hyperphosphorylation”) and aggregation of Tau protein are hallmarks of Alzheimer disease and other tauopathies, but their causative connection is still a matter of debate. Tau with Alzheimer-like phosphorylation is also present in hibernating animals, mitosis, or during embryonic development, without leading to pathophysiology or neurodegeneration. Thus, the role of phosphorylation and the distinction between physiological and pathological phosphorylation needs to be further refined. So far, the systematic investigation of highly phosphorylated Tau was difficult because a reliable method of preparing reproducible quantities was not available. Here, we generated full-length Tau (2N4R) in Sf9 cells in a well defined phosphorylation state containing up to ∼20 phosphates as judged by mass spectrometry and Western blotting with phospho-specific antibodies. Despite the high concentration in living Sf9 cells (estimated ∼230 μm) and high phosphorylation, the protein was not aggregated. However, after purification, the highly phosphorylated protein readily formed oligomers, whereas fibrils were observed only rarely. Exposure of mature primary neuronal cultures to oligomeric phospho-Tau caused reduction of spine density on dendrites but did not change the overall cell viability.

## Teaser

**Background:** The causal relationship between Tau hyperphosphorylation and aggregation in neuropathology is still under debate.

**Results:** Tau highly phosphorylated in cells increases oligomerization without pronounced aggregation. Oligomers cause reduction of dendritic spines but not cell death.

**Conclusion:** Hyperphosphorylation does not drive Tau fibrillization but contributes to synaptotoxicity.

**Significance:** Pathways and effects of Tau hyperphosphorylation are distinct from those of aggregation.

## Introduction

One hallmark of Alzheimer disease (AD)^4^ is the presence of neurofibrillary tangles in the brain, consisting of fibrillar aggregated Tau protein. Tau is a microtubule-associated protein whose binding to microtubules (MT) is regulated by kinases and phosphatases (1, 2). In AD, Tau is highly phosphorylated (up to ∼10 P_i_ per molecule) (3, 4).

The distribution of neurofibrillary tangles in the brain of patients with AD correlates highly with disease progression, which can be used to stage AD by post-mortem brain histopathology (Braak stages (5)). The causal relationship between Tau phosphorylation and neuronal dysfunction is poorly understood, but there are two main hypotheses (6). (i) A loss of function may be caused by the decreased binding of phosphorylated Tau to MT, resulting in MT destabilization and disruption of axonal transport. (ii) The toxic gain of function hypothesis argues that highly phosphorylated Tau leads to aggregation and toxic effects in neuronal cells. Recent studies of transgenic mouse models indicate that neuronal loss and memory impairment are associated with the presence of highly phosphorylated soluble Tau protein (oligomers), and suppression of its expression causes improvement in memory and an increase in the number of synaptic connections (7–9). These findings led to the concept that monomeric or low-*n* oligomeric species of Tau can cause neurodegeneration.

Whether Tau hyperphosphorylation in AD is a cause of aggregation (10) or whether the two changes occur independently is still controversial. Although phosphorylation of Tau at given sites can result in the loss of certain Tau functions (*e.g.* MT binding), the increase in phosphorylation *per se* is not necessarily detrimental, as it occurs also naturally. Tau shows a physiologically elevated level of phosphorylation in fetal mammalian brain (11, 12); Tau is transiently hyperphosphorylated during hibernation (13); and Tau shows increased phosphorylation on several sites in freshly prepared adult human and rat brains (11, 12). Moreover, Tau expressed in dividing cells acquires a number of phospho-epitopes during mitosis and is localized on spindle MTs (14, 15).

The extent of phosphorylation also differs between fetal Tau (∼6 phosphates per molecule of Tau (16)), adult cytosolic Tau (∼2 P_i_), and Tau from PHFs of AD patients (∼9 phosphates) (3, 4, 17). This makes it difficult to determine the relevant combination and extent of phosphorylation that could eventually provoke aggregation in neurons.

The quantification of phosphorylation is a challenge in studying the relationship between phosphorylation and aggregation, but this problem becomes even more complex by 85 potential phosphorylation sites (Ser, Thr, and Tyr). This equals ∼20% of the protein residues, most of which have an unknown function (if any) and only half of which (45) have been observed experimentally (18).

Tau is targeted by many kinases and phosphatases, and therefore it has been difficult to induce states of high phosphorylation and characterize their aggregation *in vitro* and in cells. One solution is the generation of phospho-mimicking mutants (*e.g.* converting Ser or Thr residues into Glu or Asp). This approach is a useful tool in Tau analysis and supports the view that there is no straightforward causal relationship between phosphorylation and aggregation (19). However, the problem remains that only a subset of P-sites can be studied and that Glu or Asp is not the perfect substitute of genuine phospho-residues (20). Another common experimental approach was to modify Tau with select kinases, determine the affected residues (using phosphorylation-sensitive antibodies or mass spectrometry), and test the aggregation of the modified protein *in vitro*. In such cases, the number of affected residues and their extent of modification were usually ill-defined (but typically much lower than that of AD-Tau), and not surprisingly, the interpretation of results remained ambiguous (21–24).

We therefore searched for alternative methods to induce a state of high Tau phosphorylation that would allow one to analyze the effects on aggregation. We took advantage of the observation that Tau can be expressed in high yield in insect Sf9 cells using the baculovirus expression system (25–27). We show here that this Sf9-Tau is in an exceptionally high state of phosphorylation and that this expression system offers a reproducible method of preparing human phospho-Tau isoforms. The biochemical and biophysical analysis of Sf9-Tau shows that hyperphosphorylation is not a direct driving force for fibril formation.

## EXPERIMENTAL PROCEDURES

### 

#### 

##### Materials

All chemicals were obtained from Sigma, Fluka (Seelze, Germany), and Roth (Karlsruhe, Germany) in highest purity if not stated otherwise.

##### Cells and Viruses

Sf9 cells were obtained from Invitrogen and grown at 27 °C in monolayer culture with Grace's medium (Invitrogen) supplemented with 10% fetal bovine serum and 50 μg/ml gentamycin and 2.5 μg/ml amphotericin. Sapphire^TM^ baculovirus DNA was obtained from Orbigen/Biozol (Eching, Germany), and pVL1392 was from Invitrogen.

##### Baculovirus Construction

The hTau40 cDNA, the longest Tau isoform in human CNS (2N4R), was excised from the bacterial expression vector pNG2 (28) with XbaI and BamHI and inserted into the baculovirus transfer vector pVL1392. For the construction of Tau containing baculovirus vectors, Sapphire^TM^ baculovirus DNA was used for homologous recombination with pVLhtau40 plasmid in Sf9 cells.

##### Protein Preparation and Purification

Sf9 cells were infected with recombinant virus at a multiplicity of infection of 1–5, typically in six T150 cell culture flasks containing 75% confluent Sf9 cells. Cells were incubated for 3 days at 27 °C and collected directly for preparation of phosphorylated hTau40 protein in lysis buffer (50 mm Tris-HCl, pH 7.4, 500 mm NaCl, 10% glycerol, 1% Nonidet P-40, 5 mm DTT, 10 mm EGTA, 20 mm NaF, 1 mm orthovanadate, 5 μm microcystin, 10 μg/ml each of protease inhibitors leupeptin, aprotinin, and pepstatin) in a ratio of 1 g of Sf9 pellet to 10 ml of lysis buffer. These procedures yielded “P12-Tau” (see “Results”). To increase the phosphorylation even further, Sf9 cells were treated for 1 h with 0.2 μm okadaic acid (OA; a phosphatase inhibitor, Enzo-LifeScience) and added to the cell medium 1 h before harvesting. Subsequently, the cells were crushed once in a French press. Next, the cells were resuspended in lysis buffer and boiled in a water bath at 100 °C for 10 min. By this treatment nearly all proteins were denatured and precipitated, except for Tau which stays soluble. The cell debris was removed by centrifuging the lysate for 15 min at 16,000 × *g,* and the supernatant containing soluble Tau protein was concentrated in Millipore Amicon Ultra-4-centrifugal filter units (molecular mass cutoff of 3 kDa). This procedure yielded “P20-Tau.”

To estimate the protein concentration in cells, we determined the OD (for *Escherichia coli* cells) by comparing the OD values with given cell numbers by Refs 29, 30 or, respectively, the number of Sf9 cells by a Neubauer counting chamber. The protein amount produced in a determined number of cells was loaded onto SDS-PAGE for Western blot analysis and estimated additionally by a bicinchoninic acid test (BCA, Sigma). This amount of proteins was then used to estimate the concentration in an average cell.

##### Size Exclusion Chromatography

The concentrated material was applied to a size exclusion column Superdex G200 (GE Healthcare) and eluted with PBS buffer (pH 7.4; 1 mm DTT), collecting 1-ml fractions. For further experiments, the fractions containing Tau protein were pooled and concentrated 10-fold to ∼50 μm. For some experiments, the concentrated protein was exchanged to BES buffer (BES 20 mm, pH 7.4 supplemented with 25 mm NaCl) using Amicon filter units (molecular mass cutoff of 3 kDa).

##### Anion Exchange Chromatography

A second purification step was performed, using anion exchange chromatography on a Mono Q HR 16/10 column (GE Healthcare). For this purpose, the Tau-containing fractions of the G200 column were pooled and dialyzed against buffer A (100 mm MES, pH 6.8, 2 mm DTT, 1 mm NaEGTA, 1 mm MgSO_4_, 0.1 mm PMSF), before loading onto the Mono Q column. Tau protein was eluted by a three-step salt gradient (buffer A supplemented with 1 m NaCl was used to create salt gradient steps of 0–0.2, 0.2–0.3, and 0.3–1 m NaCl). The protein concentration of the fractions after this purification was between 5 and 10 μm. The fractions did not contain detectable aggregates.

##### MALDI-TOF Analysis

To analyze the global phosphorylation level, hTau40 (2N4R) expressed in *E. coli* or Sf9 cells in PBS buffer was mixed with matrix solution (10 mg/ml 2,5-dihydroxibenzoic acid and 2-hydroxy-5-methoxybenzoic acid: 9 parts 2,5-dihydroxibenzoic acid in 0.1% trifluoroacetic acid with 20% acetonitrile and 1 part 5-methoxysalicylic acid in 50% acetonitrile) to yield a final concentration of 10 μm, pipetted onto a steel target plate, and dried by air. Analysis was performed on a Voyager System 4217 instrument (Applied Biosystems) kindly made available by Dr. Rob Meijers (EMBL Outstation, Hamburg, Germany). The instrument was run in the linear positive mode with delayed extraction (accelerating voltage 25,000 V; grid voltage 94%; extraction delay time 400 ns). The number of phosphates per Tau molecule was determined from the average mass shift, measured in three independent experiments.

To analyze the distribution of phosphorylation sites within Tau molecules, tryptic peptides were generated and analyzed by MALDI-TOF-MS (Analytics Laboratory, University of Giessen). The instrument was operated in the positive-ion and negative-ion reflectron mode using 2,5-dihydroxybenzoic acid and methylene diphosphonic acid in 0.1% TFA as matrix. Sum spectra consisting of 300–500 single spectra were acquired. For instrument control, data processing, and data analysis the Compass 1.3 software package consisting of FlexControl 2.4, FlexAnalysis 3.0, BioTools 3.0 (Bruker Daltonics), was used. MALDI-TOF-MS was performed on an Ultraflex I TOF/TOF mass spectrometer (Bruker Daltonics, Bremen, Germany) equipped with a nitrogen laser and a LIFT-MS/MS facility.

##### Database Search

Most of the phosphopeptides were identified by MASCOT peptide mass fingerprint search (MatrixScience, London, UK) using the Uniprot database. The search was restricted to *Homo sapiens* with a mass tolerance of 75 ppm, carbamidomethylation of cysteine as global, and oxidation of methionine and phosphorylation of serine or threonine as variable modifications, and one missed cleavage was allowed. Several phosphopeptides with multiple phosphorylation sites were assigned manually with less stringent restrictions (two missed cleavages allowed, phosphorylation of tyrosine as variable modification).

##### CD Spectroscopy

All measurements were carried out with a J-810 CD spectrometer (Jasco) in a Hellma 110-QS cuvette with a path length of 0.1 cm. The parameters were as follows: scanning speed, 100 nm/min; bandwidth, 0.1 nm; response time, 4 s; measurement temperature, 20 °C. In each experiment, 15 spectra were summed and averaged. Tau protein was measured at 1 μm in 50 mm sodium phosphate buffer, pH 6.8, and molecular ellipticity was calculated by the Jasco program Spectra analysis.

##### Sarkosyl Extraction of Aggregated Tau

Extraction of Sarkosyl-insoluble Tau from Sf9 cell culture was performed following a modified protocol for brain tissue (31). Briefly, the Sf9 cells were harvested, resuspended in buffer H (10 mm Tris-HCl, 1 mm EGTA, 0.8 m NaCl, 10% sucrose, pH 7.4) at a ratio of 1 mg of cells in 100 μl of buffer H, homogenized, and centrifuged. The supernatant was collected, and the resulting pellet was homogenized again in buffer H and centrifuged at 27,000 × *g* for 20 min at 4 °C. Both supernatants were combined, adjusted to 1% (w/v) *N*-lauroylsarcosine, and incubated with shaking at room temperature for 1 h. After centrifugation at 100,000 × *g* for 35 min at 20 °C, the pellet was resuspended in 50 mm Tris-HCl, pH 7.4, using 0.5 μl of buffer H per 1 mg of initial cell pellet. Western blotting was used to analyze the supernatant and the insoluble pellet. The ratio between the volumes of the supernatant and the Sarkosyl-insoluble pellet loaded in 10% SDS-PAGE was 30:1 (*i.e.* the amount of protein in the insoluble fraction was 30 times increased compared with the soluble fraction). For quantification of Tau levels, Western blots were probed with pan-Tau antibody K9JA and analyzed by densitometry.

##### Cell Culture

Primary hippocampal or cortical neurons were isolated from embryonic E16 mice and plated on poly-d-lysine-coated (0.05 mg/ml) glass coverslips for immunofluorescence or on coated plastic wells (24-well plates; Corning Glass) for viability assays at a density of 50,000 cells per well (for cortex, respectively, 75,000 cells per well for hippocampal cells). The plating medium was minimum Eagle's medium supplemented with 10% horse serum, 1% glucose, 500 mm pyruvic acid, and 1% penicillin/streptomycin. The medium was changed to Neurobasal medium (Invitrogen) supplemented with penicillin/streptomycin and B27 (Invitrogen) and 2 mm glutamine (PAA Laboratories GmbH) after 2–4 h, and the medium was doubled. After 96 h of incubation, the cells were treated with 300 nm cytosine β-d-arabinofuranoside (AraC, Sigma) to reduce glial growth. For experiments, the cells (hippocampal or cortical neurons) were treated at 20 days *in vitro* with 1 μm final protein concentration (Tau or Aβ) for 3 h, and then applied to the conditioned cell culture media. Aβ oligomers were aggregated *in vitro* for 1 h by mixing Aβ(1–40) and Aβ(1–42) in a ratio of 1:7 in PBS buffer following Ref. 32. Aggregation of Sf9-Tau protein was monitored by TCSPC (see below) before applying the oligomer-containing mixture to the cells.

##### Cell Viability and Cytotoxicity Assays

Lactate dehydrogenase (LDH) release was measured with the cytotoxicity detection kit (LDH; catalog no. 11 644 793 001) from Roche Diagnostics as recommended by the manufacturer. 2% Triton X-100 incubated for 3 h was used as positive control and normalized to 100% cytotoxicity. Results are shown as relative cell cytotoxicity in percent compared with buffer-treated controls (normalized to 100%) (*n* = 6; error bars indicate S.D.). The MTT assay of the cell viability kit was obtained from Roche Diagnostics (catalog no. 11 465 007 001) and used following the manufacturer's instructions. MTT reduction was measured via OD after incubating primary neuronal cortex cells with the Tau protein (1 μm) or BES buffer for 3 h in conditioned media. 2% Triton X-100 was used to induce cell death for a positive control, and the OD value of BES buffer-treated cells was set to 100%, indicating viable cells (*n* = 6; error bars indicate S.D.).

##### Immunofluorescence Staining for Actin-enriched Protrusions (Spine Counting)

Cells were grown on coverslips and fixed in 3.7% formaldehyde at 4 °C overnight and permeabilized for 5 min with 0.5% Triton X-100. Actin staining was performed by incubating the fixed cells for 1 h with phalloidin-rhodamine dye (Cytoskeleton Inc., 1:100), and cell nuclei were stained with Hoechst (Sigma). Actin-enriched protrusions were visualized using the red channel and settings (Cy3) of the cell observer Axiovert 200 M microscope (Zeiss) and a ×63 objective. Actin-enriched protrusion (“spines”) were counted on dendrites at 30 μm distance from the cell soma and over a length of 20–30 μm. For each condition, 20 cells were evaluated (from two coverslips) (*n* = 20; error bars indicate S.E.).

##### Dephosphorylation of Sf9-Tau

Phosphorylated Sf9-Tau was incubated with 10 units of calf intestine alkaline phosphatase (New England Biolabs, catalog no. M0290) per 1 μg of protein for 24 h at 37 °C in NEB-3 buffer (New England Biolabs). The phosphatase was removed by high salt treatment (5 m NaCl at 70 °C), followed by centrifugation and dialysis to PBS for MALDI analysis. Dephosphorylation during TCSPC experiments was achieved by incubation of Sf9-Tau with 10 units/μg protein of Fast-AP (thermosensitive alkaline phosphate from ThermoFisher Scientific catalog no. EF0651) in PBS buffer at 25 °C.

##### Labeling Sf9-Tau and P0-Tau with the Dye Alexa488

Covalent labeling of Alexa488 to hTau40 was achieved with the Molecular Probes thiol-reactive reagent Alexa Fluor 488 C_5_ maleimide (Life Technology^TM^). Briefly, 1 mg of Tau protein was incubated for 30 min in the presence of 10× volumes of tris-(2-carboxyethyl)phosphine and then mixed with a 5× molar excess of Alexa Fluor 488 C_5_ maleimide. After completing the binding reaction (3 h), the unbound dye was separated from the labeled protein using a NAP-5 column (GE Healthcare), and the ratio between fluorescence intensity and protein amount was determined to estimate the labeling efficiency (between 50 and 150%, attached to Cys-291 and/or Cys-322).

##### Fluorescence Anisotropy

Steady-state fluorescence anisotropy was measured with a FluoroLog spectrofluorometer (HORIBA) in an “L” format, by measuring the vertical (*I*_VV_) and horizontal (*I*_VH_) components of the fluorescence emission with the excitation light polarized vertically. Anisotropy was calculated according to Equation 1, where the *G* factor (*G* = *I*_HV_·*I*_HH_) corrects for the transmission bias caused by the detection unit,

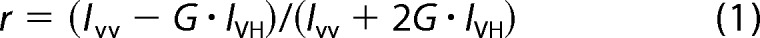
 Excitation and emission wavelengths were 490 and 515 nm, respectively, and Tau protein was covalently labeled with Alexa488. Samples were incubated in the dark at 37 °C, mixing unlabeled Tau protein (P0-Tau, P12-Tau, or P20-Tau) with labeled protein (A488-P0-Tau or A488-P20-Tau, respectively) at a ratio 10:1. Fluorescence anisotropy (*r*_ss_) was recorded at room temperature in an ultramicrocuvette (Hellma catalog no. 105.252-QS), by taking each time 30 μl out of the protein mixture.

##### TCSPC

TCSPC was carried out with a FluoroLog spectrofluorometer, using TCSPC settings and a nano-LED as a source of pulsed laser light. The light source is a periodic laser pulse, which is detected in multiple channels, making single photon counting possible (33). Excitation wavelength was 490 nm, emission wavelength 515 nm (specific for Alexa488), and the emitted light passed through a 495-nm bandpass filter. The measurement was initialized by an impulse of the specific wavelength, leading to the excited condition of the fluorophore that lasted for several nanoseconds before light was emitted. This time was measured as average lifetime of the fluorophore. The molecule's environment affects its lifetime; for example, two adjacent molecules cause quenching effects and hence reduce the fluorophore's lifetime. The fluorescence lifetime of Tau protein-bound fluorophore was expected to decrease with increasing molecular interactions during aggregation. For these experiments, the labeled A488-P0-Tau was mixed with unlabeled P0-Tau, P4-Tau, or P20-Tau in a ratio of 1:10 and incubated at 25 °C in PBS buffer. A scattering silica solution (Ludox®, Sigma) was used to measure the instrument response function. Measurements were taken every 60 min determining the average lifetime of the fluorophore. Lifetime calculations were performed with the DAS6.6 decay analysis software (HORIBA). The average weighted lifetime values (τ) for the A488-P0-Tau were determined using Equation 2,

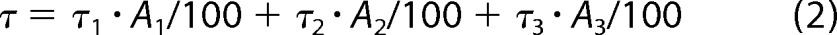
 The fluorescence lifetime components (τ_1_, τ_2_, and τ_3_) and their fractional amplitudes (A1, A2, and A3 in %) were obtained by fitting the fluorescence intensity decays of the dye to the minimal number of exponential terms that produces randomly distributed residuals (smallest error).

##### Transmission Electron Microscopy

Electron microscopy of negatively stained Tau aggregates was performed using 600-mesh carbon-coated copper grids. The samples were incubated for 3 min on a glow-discharged grid, and then the solution was removed by filter paper. Three washing steps with double distilled H_2_O (grid on top of each drop) were followed by staining with 2% (w/v) uranyl acetate for 90 s and a final short washing step with double distilled H_2_O. The grids were analyzed by a Philips CM12 electron microscope at 100 kV. In the case of imaging microtubules, they were stabilized against disassembly by adding 2% (v/v) glycerol (final concentration) and carrying out all steps at 37 °C. The RB buffer, used for the washing steps, contained also 2% glycerol. Formvar carbonated copper grids (200 mesh) were obtained from Quantifoil and glow-discharged for 30 s in a Pelco easiGlow instrument. These grids were analyzed at 200 kV by a JEOL JEM-2200FS transmission electron microscopy.

##### Atomic Force Microscopy (AFM)

For AFM measurements of aggregation time courses, 3.3 μm Tau proteins were incubated in PBS buffer at room temperature (Sf9-Tau) or in BES at 37 °C (Sf9-Tau previously concentrated to 50 μm). After 0, 24, 50, 72, and 216 h, 15 μl of Tau incubation mixture were adsorbed onto a freshly cleaved mica surface for 15 min. Excess Tau protein was removed by exchanging buffer five times against imaging buffer (PBS), and AFM height images (1 × 1 μm, 512 × 512 pixels) of P12-Tau or P20-Tau were recorded at randomly selected surface positions in peak force mode applying a contact force of 100 piconewtons, an amplitude of 20 nm, and scan rates of 1–1.2 Hz using a Nanoscope V (Digital Instruments, Santa Barbara, CA). AFM images were processed using the flattening function of Nanoscope microscope software, and the height of Tau aggregates, corresponding to aggregate diameter, was analyzed using (free software from National Institutes of Health). To determine the surface area covered by different particles, height constraints were applied to classify Tau aggregates as monomers (maximum height = 0.2–1.8 nm), oligomers (maximum height = 3.0–30.0 nm), and polymers (height ≥5.0 nm and aspect ratio ≥2).

##### Light Scattering Assay

The aggregation status of Tau samples was monitored by 90° angle light scattering at 350 nm in an ultramicrocuvette. The scattering of the buffer solution was subtracted from each value.

##### Turbidity Assays

Tau-induced microtubule assembly was monitored by 90° angle light scattering at 350 nm in a FluoroMax spectrophotometer (SPEX). 25 μm PC-purified tubulin were mixed with 5 μm Tau protein in RB buffer (100 mm PIPES, pH 6.9, 1 mm DTT, 1 mm MgSO_4_, 1 mm EGTA, 1 mm GTP). The polymerization was started by transferring the ice-cold tubulin/Tau solution to the 37 °C warm cuvette holder at time point 0 min.

##### Statistics

**FIGURE 1.**
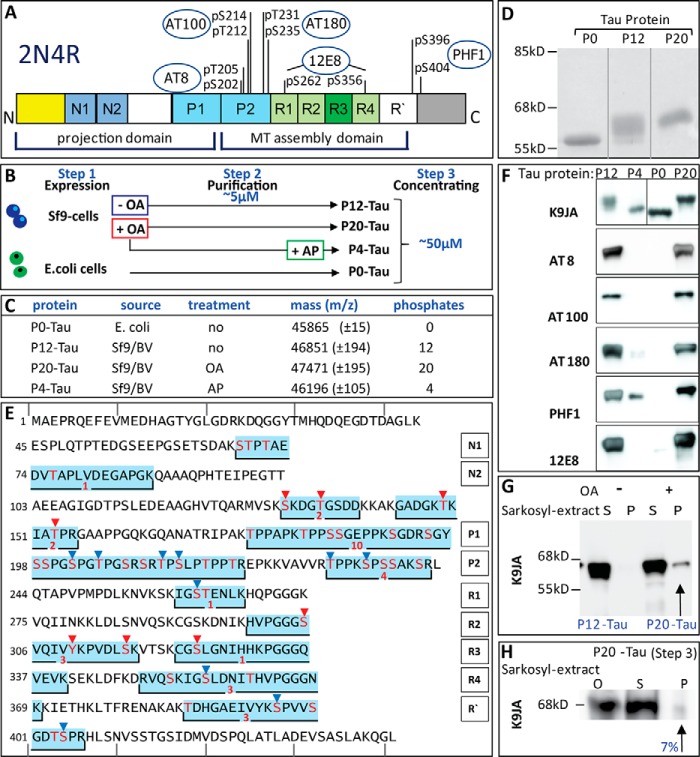
**High phosphorylation state of Tau after expression in Sf9 cells similar to AD Tau.** *A,* diagram of domains of hTau40 (2N4R) and phosphorylated sites recognized by phospho-specific antibodies. The largest human isoform of CNS Tau, 2N4R, consists of 441 residues with the three alternatively spliced inserts N1, N2, and R2. The N-terminal half represents the projection domain, and the C-terminal half constitutes the microtubule assembly domain. The core of this domain contains four repeats (R1 to R4). Epitopes of phosphorylation-dependent antibodies used in *F* are indicated in *circles. B,* Tau protein purification provides two sets of experimental material in different “aggregation” status. *Step 1,* hTau40 is expressed in Sf9 cells or in *E. coli* and reaches estimated intracellular Tau concentrations of ∼230 μm (Sf9) or ∼200 μm (*E. coli*). Sf9 cells treated with okadaic acid before harvesting (*red box*) generated P20-Tau, Sf9 cells lysed without OA treatment P12-Tau (*blue box*). *Step 2,* hTau40 is purified by size exclusion chromatography and ion exchange chromatography; anion exchange chromatography was used for Sf9-Tau protein. The resulting pooled fractions (concentrations ∼5–10 μm) were buffered in PBS, pH 7.4. At this step, the hyperphosphorylated Sf9-Tau protein already showed a higher light scattering than the unphosphorylated P0-Tau from *E. coli*. Protein was taken for light scattering experiments: TCSPC, AFM, and dephosphorylation (to P4-Tau by alkaline phosphatase). According to AFM, no oligomers are detectable at this step of preparation. *Step 3,* pooled hTau40 protein was first concentrated (to ∼50 μm) in PBS and subsequently re-buffered to BES buffer, pH 7.4. During this process some initial seeds of aggregation (nuclei) occurred in Sf9-Tau (P12 or P20) but not in P0-Tau. The concentrated protein from *step 3* was used for AFM, light scattering, fluorescence anisotropy, MT assembly, MALDI analysis, and cell culture experiments. Note that for the covalent labeling of Tau with the Alexa488 probe, concentrated protein was used to increase the efficiency of the labeling. *C,* characterization of the phosphorylation state of Tau by MALDI-TOF. The numbers of phosphates per Tau molecule were calculated by comparing the mass differences of hTau40 WT (441 amino acids; theoretical value 45,849.91 Da) expressed in *E. coli* (named P0-Tau) to Sf9-hTau40 protein (80 Da per phosphate group). Expression of hTau40 in the Sf9/BV system increased its mass by the equivalent of 12 phosphates per molecule (named P12-Tau). Treatment of Sf9 cells with OA for 1 h prior to harvesting was used to reach a status of 20 phosphates per molecule (P20-Tau). Dephosphorylation of purified P12-Tau by incubation with alkaline phosphatase (*AP*) reduced the protein to the mass equivalent of a Tau molecule containing four phosphates (P4-Tau). The average mass of the phosphorylated protein was determined in three independent experiments; the *error* is represented as S.E. *D,* SDS-PAGE analysis showed an upward shift in molecular mass with increasing phosphorylation, from 55 kDa (P0-Tau) to 68 kDa for P20-Tau. This shift is characteristic for AD-Tau. *E,* sequence of Tau (2N4R, 441 residues) showing the putative phosphorylation sites (*red letters*) in peptides obtained by in-gel trypsin digestion and subsequent MS analysis of purified P12-Tau and P20-Tau. Regions of the sequence, identified as phosphorylated peptides by mass spectrometry (*MS*), are highlighted by a *blue box* (overlapping peptides were combined). The *red number below* each box summarizes the (overall) number of identified phosphates in those peptides (for details see Table 1). Residues with an explicit identified phosphorylation site are indicated by *red triangles* (identified by MS) or by *blue triangles* when identified by combined MS analysis and phosphorylation-specific antibodies (see also *F*). The Tau domains are listed on the *right,* and the numbers of their first residues are shown in each line on the *left*. Lines on *top* and *bottom* are marking every 10th residue. *F,* phosphorylated sites of Sf9-Tau (40 ng/lane) were detected by Western blot analysis using Alzheimer diagnostic antibodies as follows: K9JA (control), a pan-Tau antibody independent of phosphorylation, which recognizes the repeat domain plus C-terminal tail (Gln-244 to Leu-441); AT8 (Ser(P)-202 + Thr(P)-205); AT100 (Thr(P)-212 + Ser(P)-214); AT180 (Thr(P)-231 + Ser(P)-235); PHF1 (Ser(P)-396 + Ser(P)-404); and 12E8 (Ser(P)-262/Ser(P)-356) (accounting for 10 different sites). 40 ng of Tau protein (P0-Tau, P12-Tau, or P20-Tau) were loaded on 10% SDS-PAGE. Note that the phosphorylation-dependent antibodies do not recognize phosphate-free protein P0-Tau. P4-Tau, the dephosphorylated product of P12-Tau, still shows weak signals at PHF1 and AT180 epitopes. *G,* highly phosphorylated Tau proteins contain only negligible amounts of aggregates in Sf9 cells detectable by Sarkosyl extraction (0.35% in case of P20, see *arrow*). *S,* supernatant; *P,* pellet. Note that the pellets were concentrated 30-fold compared with the supernatant fraction. *H,* Sarkosyl extraction of previously aggregated P20-Tau yielded only small amounts of Sarkosyl-insoluble Tau aggregates. 50 μm P20-Tau was incubated for 3 days at 37 °C in BES buffer (*step 3*) and then the Sarkosyl extraction was performed. P20-Tau showed only 7% of Sarkosyl-insoluble Tau (densitometry analysis), representing a small fraction of detergent-stable aggregates. Note that supernatant (*S*) and pellet (*P*) were both loaded at the same concentration (1:1).

**FIGURE 2.**
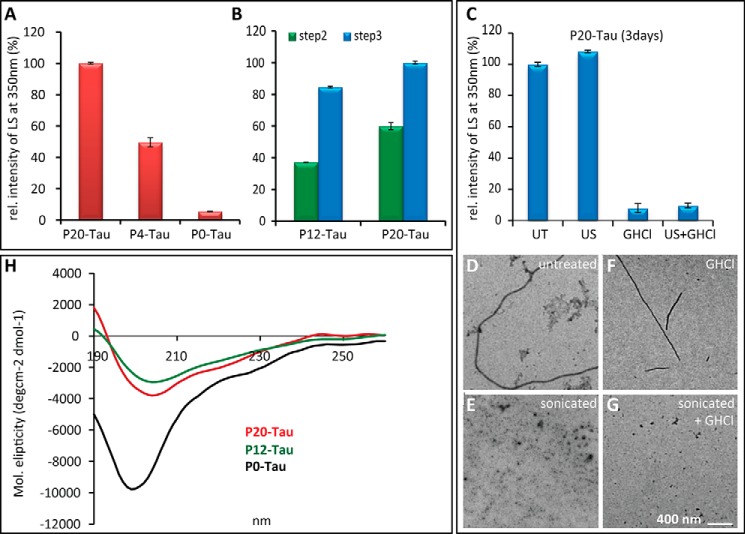
**Light scattering at 350 nm indicates increased aggregation of Sf9-Tau compared with *E. coli*-Tau.** *A,* light scattering at 350 nm of different Tau preparations in the respective buffers (PBS, BES, pH 7.4). P20-Tau was measured after concentrating in BES (*step 3*) and P0-Tau after concentrating in PBS (*step 3*). All proteins were diluted for measurements to 7 μm. P4-Tau was generated by treating P12-Tau with alkaline phosphatase (24 h, 37 °C), and alkaline phosphatase was removed before measurements. Values were normalized to buffer and to the aggregated P20-Tau in BES (100%). *Error bars* indicate the S.D. *B,* light scattering of Sf9-Tau in PBS, normalized to P20-Tau, concentrated in BES (100%). Sf9-Tau purified in PBS (*step 2*) showed a lower scattering at 350 nm than the same proteins concentrated in BES (*step 3*). P20-Tau shows in both buffers higher scattering signals than P12-Tau, measured at the same protein concentrations of 7 μm. *C,* light scattering intensity of P20-Tau (incubated at 50 μm for 3 days, 37 °C in BES buffer) was measured at 10 μm protein concentration from untreated samples (*UT*) and after treatment with 6 m guanidine HCl for 10 min (*GHCl*), respectively, and 2 min by ultrasonication (with 40% amplitude and 10-s pulses) or both treatments (guanidine HCl + ultrasonification (*US*)). Intensities are normalized to 100% (untreated P20-Tau), and *error bars* indicate S.D. (*n* = 3). *D–G,* representative electron micrographs of the light scattering samples measured in *C. D,* untreated P20-Tau revealed amorphous aggregates, next to a few fibrils. *E,* sonicating the sample removed any visible fibrils, *G,* similar to a combined treatment with guanidine HCl treatment. *F,* denaturating conditions (guanidine HCl) removed amorphous aggregates and left only some fibrils visible. *H,* CD spectra of P0-Tau, P12-Tau, and P20-Tau did not reveal substantial changes in secondary structure of Sf9-Tau. Circular dichroism spectra were recorded from 190 to 260 nm of P12-Tau (*green*), P20-Tau (*red*), and P0-Tau (*black*) at 1 μm protein concentration. The Tau protein was purified in PBS (but not concentrated in BES buffer). For the measurements, it was diluted to 1 μm in sodium phosphate buffer, pH 6.8. Sf9-Tau showed a slight red-shift of the intensity minima in the spectra at 204 nm, compared with P0-Tau at 198 nm, which represents a mostly random coil structure.

**FIGURE 3.**
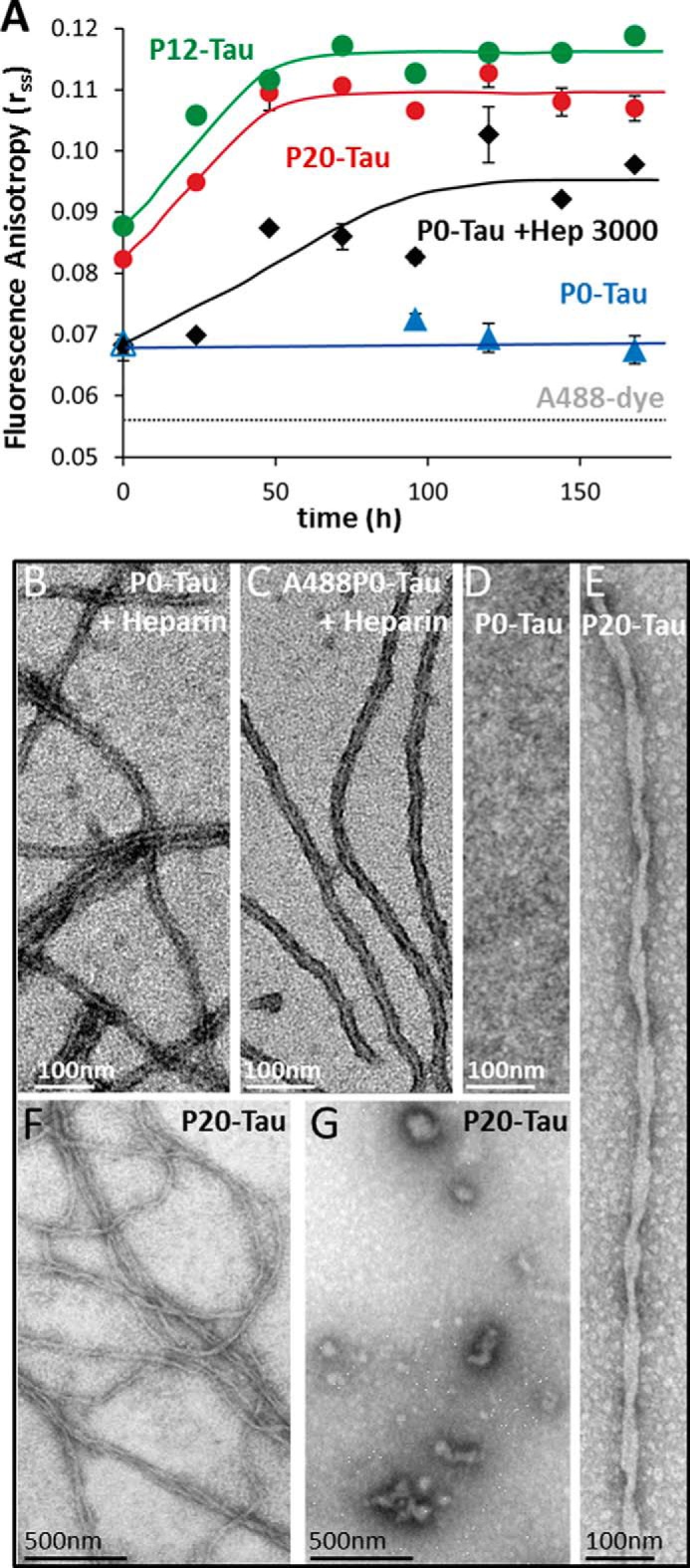
**Steady-state fluorescence anisotropy (*r*_ss_) demonstrates that high phosphorylation of Tau increases its ability for aggregation into oligomers and fibers.** *A,* aggregation kinetics of highly phosphorylated Tau measured by steady-state fluorescence anisotropy. Phosphorylated P20-Tau (*red dots*), P12-Tau (*green dots*), and P0-Tau (*black* and *blue*, control) were labeled at Cys-291 and Cys-322 with Alexa dye A488. 50 μm Tau (consisting of 45 μm native and 5 μm A488-labeled-Tau) were incubated in 20 mm BES buffer, 25 mm NaCl, pH 7.4, at 37 °C for 7 days. The increase in *r*_ss_ reflects slower depolarization of fluorophore emission due to slower Brownian motion, indicating the increase in molecular size. In the absence of heparin, P12-Tau and P20-Tau showed an increase of *r*_ss_ over time, indicating faster aggregation, compared with P0-Tau, staying at the initial level. In the case of 50 μm P0-Tau, incubated in BES buffer at 37 °C with 12.5 μm heparin (*black diamonds*), there is a significant increase of the *r*_ss_ value over time, illustrating the aggregation-inducing effect of heparin. The *r*_ss_ value = 0.056 of the free fluorophore A488 is indicated by the *dashed gray line*. The lines connecting the measurement points are eye-guided lines, and the *error bars* indicate S.E. (*n* = 3). *B–D,* negative-stain electron micrographs of 50 μm P0-Tau (*B*) or 50 μm (1:1) A488-labeled P0-Tau (*C*), incubated with 12.5 μm heparin for 240 h, illustrates the ability of unlabeled or labeled protein to form fibers of similar appearance. By contrast, no aggregates develop without heparin (*D*). *E–G,* negative-stain electron micrographs of P20-Tau (in absence of heparin) were prepared directly after concentrating the protein to 50 μm in BES buffer. Electron micrographs demonstrate some amorphous Tau aggregates (*F* and *G*), *i.e.* heterogeneous oligomers and polymers (∼7% of total protein as judged by the Sarkosyl extraction method). This includes occasional twisted fibrils of aggregated P20-Tau (*E*) with a crossover distance of ∼80 nm, similar to AD-PHFs.

Data are presented as mean ± S.E. (Figs. 1*C* and 7*C*) or S.D. (Figs. 2, *A–C*, 3*A,* and 7, *A* and *B*), respectively. Differences between mean values were determined by analysis of variance test with Tukey's least significant difference corrections for post hoc comparisons (Fig. 7*C*).

## RESULTS

### 

#### 

##### Highly Phosphorylated Human Tau Expressed in Sf9 Cells Reveals AD-like Characteristics

As a source of phosphorylated Tau (in this study the longest human CNS isoform hTau40, 2N4R, see Fig. 1*A*), we used the Sf9/BV cell system. This allows efficient expression of Tau, up to 50 μg/10^6^ cells (∼230 μm, estimated from a typical cell volume of ∼5 pl) (26), roughly equivalent to expression of other proteins (*e.g.* tyrosine kinase Abl, ∼70 μg per 10^6^ Sf9 cells (34)). By comparison, *E. coli* expresses Tau at a level of ∼9.6 μg/10^9^ cells (∼200 μm, estimated from a typical cell volume of ∼1 μm^3^ = 1 fl), equivalent to a yield of ∼9.6 mg/liters expressed protein, assuming 10^12^ cells/liter at *A*_600_ ∼1). This corresponds to an intermediate range of recombinant protein expression from *E. coli* (35). The cellular concentrations were well below the solubility of hTau40 determined *in vitro* by dynamic light scattering in a crystallization chamber (∼30 mg/ml or ∼ 650 μm for a molecular mass of 45.8 kDa). This solubility is remarkably high, consistent with the absence of inclusion bodies in both expression systems. These relationships illustrate the counterintuitive behavior of Tau protein that is highly soluble yet aggregates in pathological conditions.

MALDI-TOF analysis of *E. coli*-Tau yields a mass of 45,865 Da, in good agreement with the theoretical mass derived from the sequence (45,849.91 Da), indicating the absence of phosphorylation (termed P0-Tau). However, Sf9-Tau shows a mass of 46,851 Da, 1001 Da greater than *E.coli*-Tau, equivalent to ∼12 phosphates (80 Da each) per Tau molecule (Fig. 1*C*). Because Tau carrying 10 P_i_ per molecule is considered as abnormally phosphorylated AD-Tau (10), the Sf9-Tau fulfills the criteria of hyperphosphorylation. By incubating Sf9 cells with the phosphatase inhibitor OA directly before cell harvesting and subsequent purification of Sf9-Tau, the level of phosphorylation can be increased even further, up to 47,471 Da, *i.e.* 1621 Da greater than nonphosphorylated Tau, equivalent to ∼20 P_i_ per molecule (Fig. 1*C*). We refer to these preparations as P12-Tau and P20-Tau, respectively. Dephosphorylation of the purified phospho-Tau by alkaline phosphatase decreases the molecular mass to 46,196 Da, 346 Da greater than *E.coli*-Tau, equivalent to about 3–5 P_i_ (termed P4-Tau). This residual level of phosphorylation cannot be further reduced even by extended treatment with phosphatase. On SDS-PAGE, both highly phosphorylated Tau species (P12-Tau and P20-Tau) show a shift to 68 kDa, whereas recombinant nonphosphorylated *E.coli*-Tau (P0-Tau) runs at 55 kDa (Fig. 1*D*). This shift is characteristic of AD hyperphosphorylated Tau, also named A68-Tau (36), and was also seen for the different Tau isoforms in PHF Tau (37). The single band of P20-Tau indicates a rather homogeneous degree of phosphorylation. The P12-Tau protein displays a set of protein bands between 55 and 68 kDa.

To characterize the phosphorylation sites in more detail, we prepared tryptic peptides from purified P12-Tau and P20-Tau by in-gel digestion and analyzed them by MALDI-TOF mass spectrometry and phosphorylation-specific antibodies (Fig. 1*F*). Fig. 1*E* shows the hTau40 sequence, highlighting the identified phosphopeptides (additional data on all 19 phosphopeptides with partly overlapping sequences are listed in Table 1). The red numbers in Table 1 indicate the total number of phosphorylation sites found in a phosphopeptide, and potential phospho-residues (Ser, Thr, or Tyr) are indicated in red. Tau (2N4R) contains 85 potential phosphorylation sites (45× Ser, 35× Thr, and 5× Tyr (18)). Among these, eight sites were identified by MALDI-TOF (Fig. 1, *red triangles*) and 10 (*blue triangles*) by comparing phosphorylation-specific antibody-binding sites with the phosphopeptides (see also Fig. 1*F*). Phosphorylation sites were most prominent in the proline-rich region (P1 and P2) and the repeat domain (R1–R4), consistent with the antibody reactivity, whereas the N-terminal region of Tau revealed only few sites. Only one of the five Tyr residues appeared in phosphorylated form (Tyr-310), and the others remained undetected even though they are known targets of tyrosine kinases Fyn of Abl (*e.g.* Tyr-18 and Tyr-394 (18); note, however, that the MS method does not yield quantitative results, due to the variable behavior of the tryptic peptides in the instrument).

**TABLE 1:**
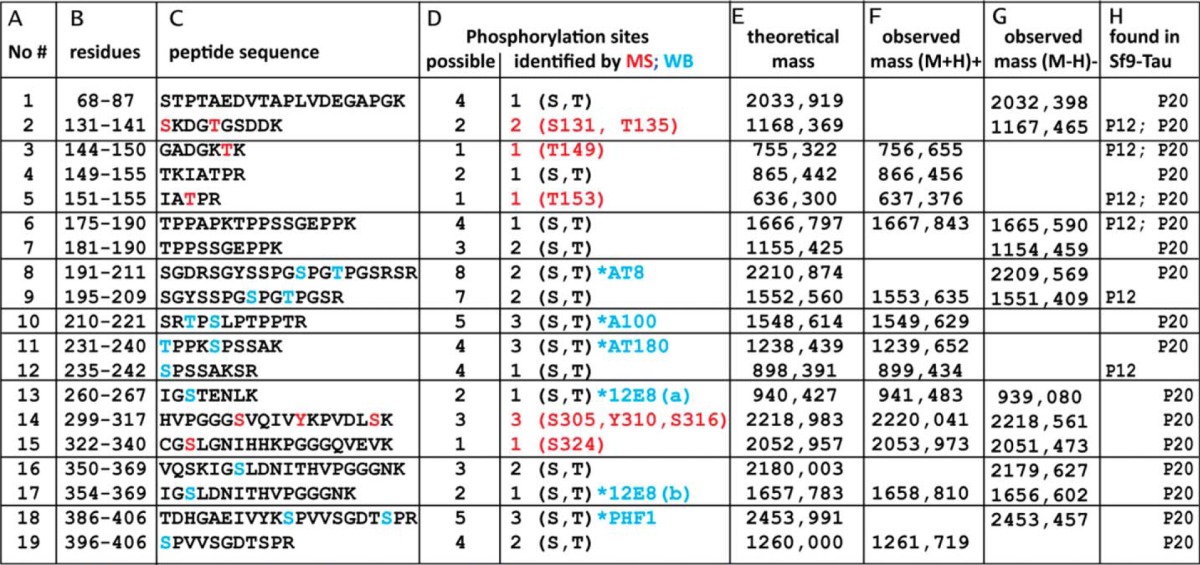
**Phosphorylated peptides of Sf9-Tau (P20 and P12) observed by mass spectrometry** Column A, tryptic peptides were obtained from P12-Tau and P20-Tau expressed in Sf9 cells and analyzed by MALDI-TOF-MS; the table lists 19 phosphorylated peptides. Column B, residue numbers of each peptide (start/end) are according to the hTau40 sequence. Column C, amino acid sequences of the identified peptides with highlighted residues are shown. Red letters are for MS-identified phosphorylated residues, and blue letters are for phosphorylation sites identified by phosphorylation-specific antibodies. Column D, possible number (left column) and number of identified (right column) phosphorylation sites in the peptides are shown. If the exact site could not be identified by MS (red) and/or Western blot (WB, blue), only the number of phosphates is given with the possible residues (S, serine; T, threonine). Column E, theoretical mass was calculated for the peptide. Column F, observed mass of peptide at the positive reflector ([M + H]^+^) is shown. Column G, observed mass of peptide at the negative reflector ([M − H]^−^) is shown. Column H lists the phosphorylated peptides found in samples of P12-Tau and/or P20-Tau.

Western blot analysis of Sf9-Tau confirmed important phosphorylation sites by AD diagnostic antibodies. The polyclonal pan-Tau antibody K9JA detects the recombinant Tau proteins from both *E. coli* and from Sf9 cells (Fig. 1*F*) and serves as a control. The AD diagnostic antibodies against Tau phosphorylation sites (AT8, AT100, AT180, PHF1, and 12E8) recognize exclusively the Sf9-Tau protein, indicating that these epitopes (outlined in Fig. 1*A*) are highly phosphorylated (Fig. 1*F*). The dephosphorylation of Sf9-Tau by alkaline phosphatase (to P4-Tau) leads to the complete loss of immunoreactivity at the epitopes of AT8, AT100, and 12E8 antibodies. By contrast, PHF1 and AT180 antibodies retain some weak signal even after phosphatase treatment (*P4-Tau* in Fig. 1*F*), suggesting that some of the residual phosphorylation is located at the AT180 and PHF1 epitopes upstream and downstream of the repeat domain (Fig. 1*A*).

##### Aggregation State of Phosphorylated Tau in Sf9 Cells and in Vitro

Despite the high cellular concentrations of Tau, its aggregation in both *E.coli* and Sf9 cells is negligible. This holds for Sf9-Tau even though it is highly phosphorylated. Sarkosyl extraction of P20-Tau from Sf9 cells after OA treatment results in a very small Sarkosyl-insoluble fraction, accounting for only 0.35% of the total extracted Tau (Fig. 1*G*), and P12-Tau (without OA treatment) contains no detectable Sarkosyl-insoluble Tau protein at all. In agreement with this, live-cell staining of Tau-expressing Sf9 cells with thioflavin S (ThS) does not display any ThS fluorescence signal (data not shown). For comparison, *E. coli*-Tau yields no Sarkosyl-insoluble Tau either, as expected for this highly soluble protein.

Because of the large charge difference between the phosphorylated and unphosphorylated Tau proteins, *E. coli*-Tau (P0, net charge = +4.8) and Sf9-Tau (P12, net charge −13.2; and P20, net charge −25.2) were purified by different chromatographic procedures. P0 was first loaded on the cation exchange SP-Sepharose column, followed by separation via the Superdex 200 gel filtration column. In contrast, the highly phosphorylated Sf9-Tau was first loaded on the Superdex gel filtration column, followed by anion exchange chromatography on a MonoQ column. This part of the purification procedure, termed step 2, yields a lower protein concentration (Fig. 1*B*). Both proteins were next (step 3) concentrated from ∼5 to ∼50 μm and re-buffered from PBS to BES for experimental purposes. The purity of Tau protein was analyzed and confirmed by silver-stained SDS-PAGE, where we specially ascertained that the Sf9-protein p10 (38) was absent in the pooled fractions (data not shown).

Sf9-Tau revealed striking biophysical properties already at the low concentration of 5 μm. In this case, commonly used fluorometric methods based on dyes like ThS, thioflavin T, or 8-anilino-1-naphthalenesulfonate (39) failed to produce reliable data for monitoring the aggregation. ThS or thioflavin T dyes increase their fluorescence when interacting with proteins that undergo conformational changes or aggregate into structures rich in β-sheets (40). Nevertheless, mixing these dyes with Sf9-Tau protein led immediately to high signals, independently of protein concentration and incubation time. The likely cause of this phenomenon is an unspecific binding of fluorescent dyes to the highly phosphorylated Tau protein (analogous to effects of Tau bound to arachidonic acid micelles) (41, 42). Therefore, we applied light scattering at 350 nm as a method for monitoring the aggregation state of purified proteins in solution. P20-Tau showed strongly (10×) increased light scattering signal, compared with P0-Tau (Fig. 2*A*). Reducing the phosphorylation of Sf9-Tau by treatment with alkaline phosphatase decreased the light scattering signal to 50% (Fig. 2*A*) and also depleted the high ThS signal (after exchanging the buffer by dialysis, data not shown). The 10-fold concentration to 50 μm (step 3) of P20-Tau in BES buffer (a buffer conducive to PHF aggregation (43)) led to the appearance of a small Sarkosyl-insoluble pellet of Tau aggregates containing ∼7% of total Tau (Fig. 1*H*). This was consistent with a further increased light scattering signal, reaching 18-fold higher intensity than P0-Tau (Fig. 2*B*).

##### High Phosphorylation of Tau Promotes Formation of Oligomers and Small Aggregates

The limited information in monitoring the aggregation kinetics of phosphorylated Sf9-Tau proteins by light scattering prompted us to look for complementary spectroscopic and structural methods. We used fluorescence anisotropy to monitor the aggregation in real time, using an Alexa probe (A488) covalently attached to Tau derived from *E. coli* or Sf9 cells. The probe was linked to Cys-291 and/or Cys-322 within the repeat domain and did not inhibit the fibril formation when mixed at a low ratio (1:10) with unlabeled Tau protein. Negatively stained electron micrographs of 50 μm A488-labeled P0-Tau, incubated with 12.5 μm heparin as an assembly inducer, showed PHFs from the labeled protein alone (Fig. 3*C*), similar to fibrils from unlabeled P0-Tau with heparin (Fig. 3*B*). P0-Tau protein in the absence of heparin does not form any fibrils under the same conditions (Fig. 3*D*), consistent with its high solubility. Because the aggregation of the fluorophore-labeled Tau leads to the formation of PHF-like fibers, we utilized A488-Tau proteins (in a 1:10 ratio) for the following experiments.

Steady-state fluorescence anisotropy (*r*_ss_) serves as an indicator of the rotational motion of the fluorophore (44). A small fluorescent molecule that is freely and rapidly tumbling in solution has almost zero anisotropy. An increase in *r*_ss_ value indicates a slower rotational motion of the protein-bound A488 probe and thus a slower depolarization of fluorescence emission, reflecting the gradual immobilization within the growing aggregates. For an essentially immobile protein of 50-kDa size, labeled by A488 (*i.e.* human serum albumin in 100% vitrified glycerol), the expected *r*_ss_ value is 0.37 (45), whereas the free dye A488 has an experimental value of 0.056 (Fig. 3*A*, *dashed line*), and protein-bound A488 and aggregates have intermediate values depending on their tumbling rates in solution (typically 0.1–0.2, see Ref. 46 for an example of prion protein Sup35).

For the experiments, 45 μm unlabeled Sf9-Tau (P12-Tau and P20-Tau concentrated in BES; step 3) and 5 μm A488-labeled Sf9-Tau (10% of total Tau) were incubated in BES buffer at 37 °C. Fig. 3*A* shows the gradual increase of *r*_ss_ of the A488 probe conjugated to P20-Tau (*red dots*) and P12-Tau (*green dots*) over time. P20-Tau and P12-Tau gradually reached a plateau of *r*_ss_ ∼0.11 after 70 h of incubation, indicating the formation of aggregates, which did not further decrease their molecular motion, indicating changes in molecular size. By contrast, the phosphate-free P0-Tau (5 μm P0-Tau-A488 and 45 μm P0-Tau) barely showed any aggregation in the absence of heparin, represented by a constant and low *r*_ss_ value of ∼ 0.07 during the incubation (Fig. 3*A*, *blue triangles*). Incubating P0-Tau in the presence of 12.5 μm heparin-3000 increases the *r*_ss_ value from the initial 0.07 to 0.098 (Fig. 3*A*, *black diamonds*).

The initially higher *r*_ss_ value of P20-Tau of 0.08 might be explained by the previous treatment of the sample (concentrating in *step 3*; Fig. 1*B*), leading to the formation of oligomers and small aggregates. This included even some fibrils at the initial time point in P20-Tau (and of P12-Tau, data not shown), as confirmed by electron microscopy (Fig. 3, *E–G*) of samples concentrated in BES. Fig. 3*E* shows a twisted Tau fibril, similar to PHFs found in the brains of patients with AD. However, these fibrils (overview in Fig. 3*F*) were not the only visible structures, as the electron micrographs revealed numerous heterogeneous aggregates of smaller size as well as amorphous aggregates (Fig. 3*G*). Samples taken for electron microscopy at the final time point (160 h) of anisotropy measurements did show fibrils. Nevertheless, the fraction of stable aggregates remain low. This is indicated by the small Sarkosyl-insoluble pellet (7%; Fig. 1*H*) after 3 days of incubation, and the sensitivity to 6 m guanidinium-HCl, where the high scattering intensity of Sf9-Tau (arising from all aggregates, structured or amorphous) is almost completely lost (down to 10%; Fig. 2*C*), with only rare intact fibrils visible by EM (Fig. 2*F*). However, sonication of P20-Tau destroyed most fibrils (compare Fig. 2, *D* and *E*), although the amorphous structures maintain a high scattering intensity (Fig. 2*C*).

The secondary structure of hyperphosphorylated Tau was further analyzed by circular dichroism spectroscopy (CD). In all cases, the spectra showed a prominent negative peak around 200 nm, typical of a random coil structure, and there was little difference between partially aggregated Sf9-Tau and nonaggregated P0-Tau (Fig. 2*H*). This is consistent with an earlier experience that the CD spectrum of Tau is dominated by the disordered structure, both in the soluble and in the aggregated protein (47, 48). We conclude that independently of the degree of phosphorylation, the overall character of Tau as a natively unfolded protein is retained in Sf9-Tau. However, the positive values between 190 and 195 nm seen for both Sf9-Tau curves are highly unusual for Tau protein. It suggests a contribution from some α-helical structures, similar to some mutants of full-length Tau, *e.g.* V337M (48). In addition, a slight red shift in the spectrum was characteristic for the phosphorylated Tau, resulting in an intensity minimum at 204 nm and not at 199 nm as seen for P0-Tau. The intensity (molecular ellipticity) of Sf9-Tau was 2.5 times smaller compared with unphosphorylated Tau of the same protein concentration.

The fluorescence anisotropy demonstrated the ability of highly phosphorylated Tau to promote oligomeric or higher assemblies (amorphous globular structures, including some fibers), visible by EM after increasing the protein concentration. To characterize the particles further and to monitor their formation with time, we applied alternative methods, such as TCSPC and AFM.

##### TCSPC and AFM Reveal Oligomeric Species in Hyperphosphorylated Sf9-Tau

We next investigated the Sarkosyl-soluble structures by TCSPC and AFM. TCSPC is a fluorescence-based method where the lifetime distribution of a fluorophore serves to monitor the microenvironment of a dye. Intrinsic or covalently attached dyes on protein molecules exhibit different lifetimes depending on the state of aggregation (49, 50). The lifetime of free Alexa A488 is ∼4.1 ns (51). Covalent attachment to P0-Tau at residues Cys-291 and Cys-322 barely decreased the lifetime to ∼3.8 ns (Tau-bound). The lifetime can be altered due to quenching effects when the A488-Tau molecules interact more closely or frequently with other molecules. Thus, in the case of α-synuclein, it was possible to distinguish monomers, oligomers, and fibers on the basis of the fluorescence lifetime of bound Alexa-488 (49).

**FIGURE 4.**
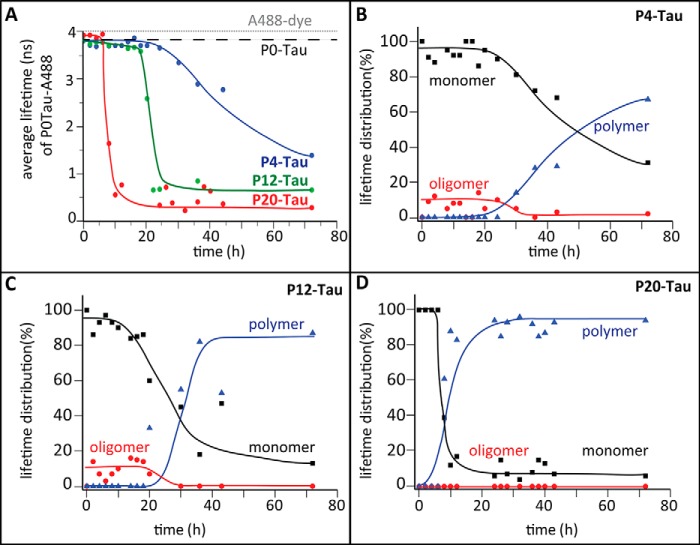
**Analysis of aggregating phosphorylated Tau by TCSPC.** *A,* average fluorescence lifetime of the A488 probe conjugated to P0-Tau (at Cys-291 and Cys-322) was measured *versus* time in a (1:10) mixture with P20-Tau (*red*), P12-Tau (*green*), or P4-Tau (*blue*). 5 μm of each protein was incubated at 25 °C in PBS buffer, pH 7.4, containing 0.5 μm A488-P0-Tau, and measurements were taken every 120 min. The decrease of the average lifetime indicates the formation of protein aggregates due to a compaction of molecules. In contrast to the anisotropy measurements (Fig. 3), the lifetime measurement also detects dynamic molecular interactions. The average lifetime of the unbound A488-fluorophore is 4 ns (*gray dashed line*), and the lifetime of monomeric P0-Tau is 3.6 ns (*black dashed line*). P12-Tau was incubated with alkaline phosphatase, resulting in P4-Tau (*blue*) (de-phosphorylation was confirmed by Western blot analysis; data not shown). *B–D,* different lifetime components were obtained by analyzing the fractional amplitude of the average lifetime τ_av_ according to τ_av_ = τ_1_·α_1_ + τ_2_·α_2_ + τ_3_·α_3_. During the time course experiments, we grouped different lifetime components as follows: monomers (3–4-ns species, τ_1_), oligomers (1–3 ns; τ_2_), and polymers (0.1–1.0 ns; τ_3_). The fractional distributions of monomers (α_1_, *black*) oligomers (α_2_, *red*), and polymers (α_3_, *blue*) are represented in % *versus* time for P4-Tau (*B*), P12-Tau (*C*), and P20-Tau (*D*). All three lifetime species add up to 1 (indicated as 100%) of the fractional amplitude. Note that the presence of oligomers within the 1st h of de-phosphorylation (*B*) reflects the initial status of P12-Tau (compare with *C*), which was incubated from 0 h onward with alkaline phosphatase.

Even without extended incubation, protein taken during the purification after concentrating the sample (Fig. 1*B*, *step 3*) shows some oligomeric and fibrillar structures in electron micrographs of P20-Tau (Fig. 3, *E* and *F*). This protein solution (50 μm at 37 °C in BES buffer) was not suitable for a kinetic study by the lifetime technique because the initial composition was already heterogeneous and showed a decreased average lifetime of ∼2.5 ns at the beginning of the experiment. To monitor aggregation kinetics from the beginning and to reduce the chance of pre-formed aggregation nuclei, we selected less concentrated protein (5 μm) from step 2 of the purification and incubated it at 25 °C in PBS. This was meant to slow down the kinetics and to enable us to detect the early events during aggregation. Accordingly, 0.5 μm Alexa488-P0-Tau was mixed with Sf9-Tau in the ratio 1:10 to monitor the lifetime upon incubation with 4.5 μm P20-Tau (Fig. 4*A*, *red curve*), P12-Tau (*green*), and P4-Tau (*blue*) over time. The 1:10 ratio of labeled and unlabeled protein was chosen to avoid energy transfer between two adjacent Alexa488-P0-Tau molecules. The average lifetime in the phosphorylated P20-Tau sample decreased rapidly after 10 h, much faster than the decrease observed for P12-Tau (∼20 h) and of the slowly decreasing lifetime in the dephosphorylated P4-Tau (50% of the average lifetime was reached after ∼50 h).

During the incubation of P12-Tau with alkaline phosphatase (to generate P4-Tau, *blue curve* in Fig. 4*A*), we monitored the average lifetime using A488-P0-Tau. In these conditions, the dephosphorylation reaches its maximal extent after 20 h. However, incubating P0-Tau (monitored by A488-P0-Tau) in the same conditions did not reveal major changes in the average lifetime (3.8 ns indicated by *black dashed line*; Fig. 4*A*), supporting earlier data of impaired Tau aggregation in absence of heparin. For comparison, the lifetime of the unbound dye shows a higher value of 4 ns without any change in lifetime (Fig. 4*A*, *gray dashed line*).

Comparing the average lifetime of these three differently phosphorylated Tau proteins indicated that a high degree of phosphorylation causes a faster transition from a high to low fluorescence lifetime of the labeled Tau. P20-Tau showed the lowest final value (0.3 ns, indicating more compact structures) than P12-Tau (0.6 ns) and P4-Tau (1.3-ns average lifetime, typical for less phosphorylated Tau). With increasing numbers of phosphates per Tau molecule, the lag time of assembly became shorter (*i.e.* the phase before the strong decrease in average lifetime, Fig. 4*A*), and more compact polymeric structures are present in the final state.

On the basis of different lifetime components observed during the time course experiments with P4-Tau, P12-Tau, and P20-Tau, we grouped the lifetime components into categories: 3–4 ns for monomers (*black squares*) of hTau40, 1–3 ns as oligomers (*red circles*), and 0.1 to 1.0 ns as polymers (*blue triangles*), as presented in Fig. 4*B*. The data were obtained by analysis of fractional amplitudes of the average lifetimes (fractional amount of particular species in the solution, the sum being 100%) of P4-, P12-, and P20-Tau (Fig. 4, *B–D*). The final average lifetime value of 0.5 ns observed for P12-Tau and P20-Tau might be caused either by a compaction in folding or to an increased frequency of fluorophore interactions. However, the instability of the Tau assemblies under high salt conditions (treatment with *GHCl*, Fig. 2*C*) and their Sarkosyl solubility (Fig. 1*H*) argue for a relatively loose packing and a highly dynamic interaction of the aggregating molecules.

During the incubation of P20-Tau the fraction of oligomers was below the detection limit, even when the fraction of polymers in the solution was already above 60% after 9 h (Fig. 4*D*). By contrast, P12-Tau revealed oligomers (*red dots*) before transformation into further aggregated structures after 30 h, with 55% of the lifetime species being polymers (*blue triangles,* Fig. 4*C*). Sf9-Tau monitored during dephosphorylation by alkaline phosphatase displayed a much slower increase of polymeric lifetime species, which started at 30 h of incubation (Fig. 4*B*). In this P4-Tau sample up to 30% of monomeric species were still present at 70 h, indicating a less compact packing (or reduced interaction) of molecules and a clearly slower process of aggregation. (Note that the presence of alkaline phosphatase did not alter the lifetime of the A488-P0-Tau (2 μm in PBS over the same incubation period). Choosing stronger aggregation conditions, using previously concentrated P20-Tau (*step 3*; Fig. 1*B*) and incubating this highly phosphorylated Tau in 50 μm BES buffer at 37 °C, we observed the formation of oligomers after 3 h. This sample contained 25% oligomeric lifetime species and was used for toxicity experiments in neuronal cells (see Fig. 7*C*).

**FIGURE 5.**
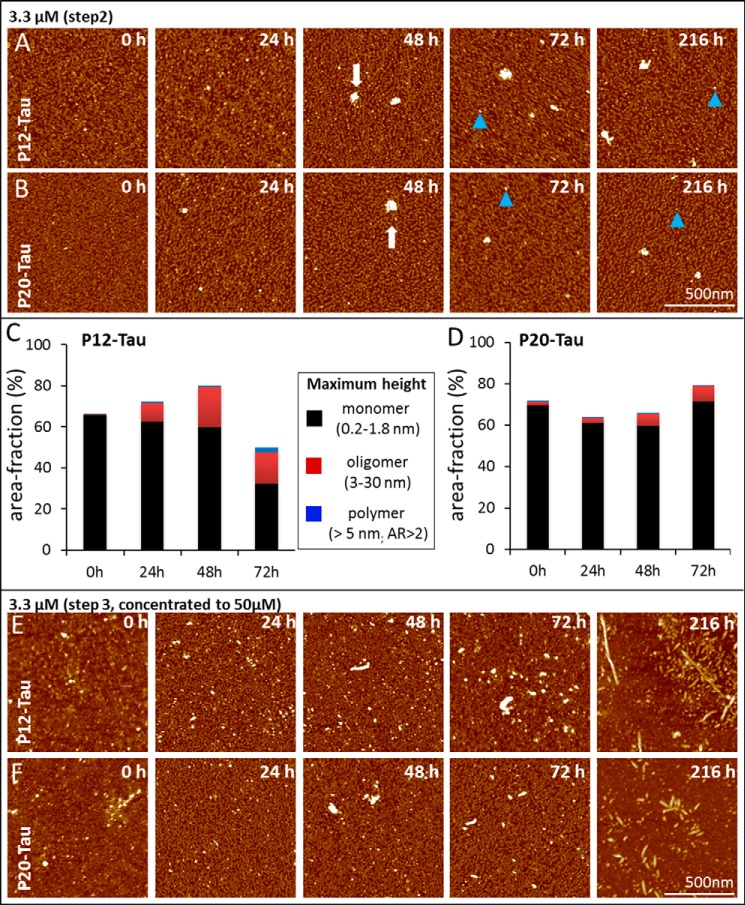
**Aggregation of phosphorylated Tau protein monitored by atomic force microscopy.** *A* and *B,* AFM visualizing the morphology of particles during aggregation. *A,* 3.3 μm P12-Tau, and *B,* 3.3 μm P20-Tau both incubated in PBS buffer at room temperature (taken from *step 2*, Fig. 1*B*). Particle sizes were obtained by measuring the maximum height of aggregates. Images of 1 square μm were randomly taken at 0, 24, 48, 72, and 216 h. Monomers between 0.2 and 1.8 nm maximum height occupy the majority of the mica surface from the start (0 h), whereas single blob-like aggregates (∼50–100 nm) are visible occasionally from 48 h on in P12- and P20-Tau (*white arrow*). (*Blue triangle* indicates oligomers with a size between 5–30 nm.) *C* and *D,* quantitative image analysis of AFM data from samples of P12-Tau and P20-Tau incubated at a concentration of 3 μm for 80 h in PBS at room temperature (see *A* and *B*). The area fraction occupied by a defined particle category was determined by image analysis software using the following parameters: monomer, maximum height range 0.2–1.8 nm; oligomer, maximum height between 3 and 30 nm; polymer, maximum height greater than 5 nm and aspect ratio >2. The fraction of the area occupied by the three aggregation species is plotted at the different time in *C* (P12-Tau) and *D,* (P20-Tau). Both Sf9-Tau proteins are dominated by monomers in the beginning (0 h), with P12-Tau showing no oligomers and P20-Tau having only 2% of the area occupied by oligomers. By further incubation, the number of oligomers increases in P12-Tau to 10% (24 h) and up to ∼20% (48 and 72 h). The presence of oligomers in P20-Tau is less prominent but increases also from 2% (at 0 h) to 7.5% at 72 h. *E* and *F,* AFM visualizing the aggregation of a previously concentrated sample of P12-Tau (*E*) and P20-Tau (*F*) was taken from purification *step 3* (Fig. 1*B*) and incubated at 3.3 μm protein concentration in BES buffer at room temperature. Both samples show aggregates of various size (monomers, oligomers, and aggregates ∼40–50 nm) at 0 h, which is a result of concentrating the protein before incubation. By further incubation, some PHFs are formed from P12-Tau after 72 h (visible with a length between 460 and 520 nm) and short rod-like fibrils (displaying a length of 50–100 nm) in the case of P20-Tau. In the final image at 216 h, mainly polymers and oligomers are visible, accompanied by few fibrils.

The fluorescence lifetime provided an accurate assessment for the transition from Tau monomers to the first molecular interaction, but it revealed little structural insight into the formed aggregates. We therefore performed AFM experiments of differently phosphorylated Sf9-Tau in a time-resolved manner (Fig. 5, *A–F*) at low protein concentration (3.3 μm). Tau protein, P12-Tau and P20-Tau, was either incubated in PBS (Fig. 5, *A* and *B*, from purification *step 2* in Fig. 1*B*) or in BES buffer (Fig. 5, *E* and *F*, from purification *step 3*, see Fig. 1*B*). Protein taken from step 3 was concentrated to 50 μm and re-buffered, and therefore it contained already small aggregates (nuclei) at the beginning (0 h, Fig. 5, *E* and *F*). This condition was chosen to monitor the formation of PHFs. P12-Tau and P20-Tau aggregated slowly at room temperature, when taken from step 2 (Fig. 1*B*) and incubated in PBS. Over time, the particles increased in height, indicating the formation of oligomers and amorphous aggregates but no fibrils (Fig. 5, *A* and *B*).

The AFM measurements taken at different time points in PBS buffer (0, 24, 48, and 72 h, Fig. 5, *A* and *B*) were quantitatively analyzed with regard to the distribution of three morphological species (Fig. 5, *C* and *D*). The observed species were categorized as monomers (maximum height between 0.2 and 1.8 nm), small oligomers (3–30 nm), and polymers (maximum height >5 nm and aspect ratio >2) on the basis of their size (height) and elongation (aspect ratio). We estimated a 30-nm spherical particle of hTau40 to contain about six molecules and therefore named it “oligomer,” whereas the additional feature of an elongated particle (with an aspect ratio of >2) implies the existence of more building units and was therefore classified as “polymer.” The distribution of these species was computed (fraction of occupied area in images; Fig. 5, *C* and *D*) for the unfavorable aggregation condition shown in Fig. 5, *A* and *B*.

At 0 h, the main structural species (occupying ∼70% of the area) present in P12- and P20-Tau are about 1–2 nm in size, roughly globular, and therefore named “monomer.” For P12, the number of monomers decreased over time, whereas the P20-Tau showed a small increase in monomers (Fig. 5*C*) at 72 h. Oligomers, defined by a size between 5 and 30 nm maximum height, appeared after 24 h in P12-Tau. Similar to the observation by lifetime measurements, P20-Tau barely showed any formation of oligomers (Fig. 5*D*). However, we could not observe an elongation of aggregated species in this condition and detected only very few polymers (Fig. 5, *C* and *D*).

Concentration of P12-Tau (to 50 μm, BES buffer) reveals already small aggregates (oligomers) at the initial time point (Fig. 5*E*, incubating at 3.3 μm). The particles further increased in size and elongated until 216 h when some twisted fibrils were visible, resembling *bona fide* PHFs. Although P20-Tau aggregated in a similar manner, the fibrils formed at 216 h were shorter and more rod-like. In contrast to Sf9-Tau, P0-Tau never formed fibrils without any inducer, and the concentration step is not sufficient to induce such aggregation (data not shown).

In summary, analysis of the influence of phosphorylation of Tau on the molecular level of interaction by combining complementary experimental approaches (fluorescence lifetime and AFM) allowed us to characterize the incipient aggregation of phosphorylated Tau. Although P20- starts faster with molecular interactions than P12-Tau (shown by an early decrease in the average lifetime), the morphology and distribution of aggregates are similar between P12-Tau and P20-Tau (mainly monomers in AFM). Concentrating the phosphorylated protein was necessary to induce nucleation, where both proteins initially showed oligomers in AFM, and after a long incubation, some of the aggregates formed into twisted fibrils (P12-Tau) and rod-like fibrils (P20-Tau). We conclude from these experiments that the hyperphosphorylation of Tau alone is not sufficient to cause fibril formation. But the increase in phosphorylation facilitates molecular interactions and mainly promotes the formation of oligomers. Additional factors such as increasing the concentration are required to result in a (small) fraction of fibers.

##### Hyperphosphorylated Tau Does Not Promote Microtubule Assembly

**FIGURE 6.**
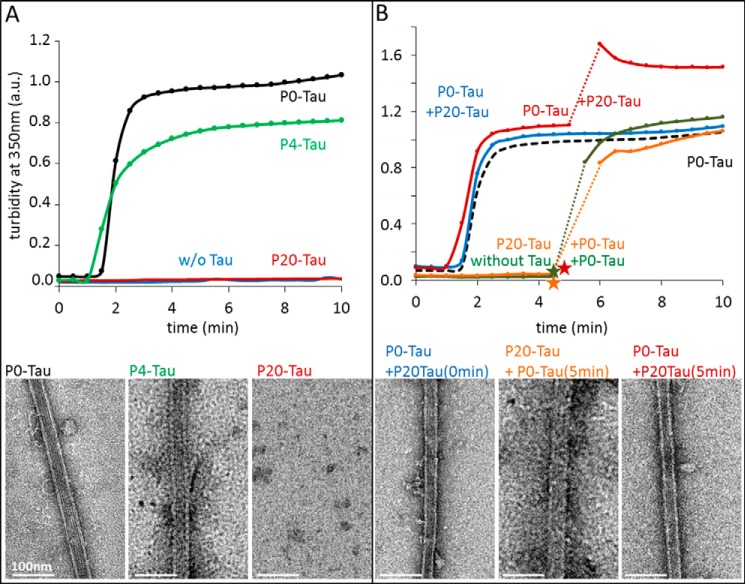
**Effect of Tau phosphorylation on Tau-induced microtubule assembly.** Light scattering at 350 nm was recorded over time to follow the microtubule assembly in the presence of Tau in different states of phosphorylation. Tubulin concentration was 25 μm; Tau concentrations were 5–10 μm, and assembly experiments were performed in the presence of 1 mm GTP at 37 °C. Directly after the turbidity experiments, the solutions were stabilized with 2% glycerol and immediately prepared for negative stain EM (kept at 37 °C during the procedure); representative images are shown *below* the curves. *A,* unphosphorylated P0-Tau (*black curve, bottom left image*) induces pronounced assembly of microtubules (from phosphocellulose-purified tubulin) with a plateau value normalized to 1. The phosphorylated P20-Tau (*red, bottom right image*) cannot promote tubulin assembly. Tubulin alone (*blue*) does not assemble in these conditions either. Dephosphorylation to P4-Tau (with alkaline phosphatase) largely rescues its MT assembly competence (*green, bottom middle image*) at 5 μm concentration. *B,* 5 μm unphosphorylated P0-Tau retains its ability to promote MT assembly in the presence of equal concentrations of 5 μm hyperphosphorylated P20-Tau (*blue, bottom left image*), indicating that P20-Tau is neutral with regard to MT assembly. P20-Tau does not sequester P0-Tau from assembled microtubules when added to initially P0-Tau-assembled MT (*red curve, bottom right image*) after 5min (time point shown by *red star*). Tubulin does not assemble with P20-Tau (*orange, bottom middle image*), but it regains assembly competence when P0-Tau is added after 5 min (*orange star*), showing that unphosphorylated Tau overrules the inert hyperphosphorylated Tau. Tubulin incubated alone (*green curve*), before adding P0-Tau (*green star*) after 5 min, shows a similar assembly (control). Curves are normalized to 1 arbitrary unit (*A.U.*) of P0-Tau and tubulin at 5 min (plateau phase), which is presented as a control as *black dashed curve*.

Besides the effect on aggregation, a second characteristic feature of hyperphosphorylated Tau is its altered binding to microtubules and the resulting physiological effects on the cytoskeleton. The main cellular function of Tau is considered to be the promotion of MT assembly and their stabilization (52). The interaction is regulated by the phosphorylation of Tau at specific sites. Hyperphosphorylation of Tau reduces its binding to MTs and could thus lead to MT breakdown. To verify the MT binding properties of phosphorylated Sf9-Tau, we compared P20-Tau and P0-Tau in an *in vitro* MT assembly assay based on light scattering at 350 nm (Fig. 6). Whereas unphosphorylated P0-Tau induced MT assembly within a few minutes (Fig. 6*A*, *black curve,* verified by electron microscopy, *bottom left image*), no MT assembly occurred in the presence of highly phosphorylated Tau (P20, *red curve,* and *bottom right image*) or without Tau (*blue curve*). MT assembly was mainly restored when using de-phosphorylated P4-Tau (Fig. 6*A*, *green curve,* and *bottom middle image*), but it still is completely impaired in the presence of P12-Tau, although it has a reduced phosphorylation status (data not shown).

Independently of the loss of Tau function (MT binding) by phosphorylation, there is a debate on the possible gain of toxic function. One hypothesis is that phosphorylated Tau might interfere with P0-Tau and sequester it away from MT, leading to their disassembly (53). We took advantage of the high phosphorylation state of Sf9-expressed Tau to investigate the possible sequestering effect. A mixture of each 5 μm P0-Tau and 5 μm P20-Tau (10 μm total Tau) was incubated for 1 h at 37 °C before the experiment, which was clearly able to support MT assembly (Fig. 6*B*, *blue curve,* and *bottom left panel*), with the same lag phase and elongation rate as 5 μm P0-Tau alone (Fig. 6*B*, *black dashed curve,* 1st 10 min). By contrast, P20-Tau alone showed no assembly activity (Fig. 6*B*, *orange curve,* until 300 s). This illustrates that highly phosphorylated Tau is inactive by itself but does not affect unphosphorylated Tau with regard to MT assembly. We next examined the influence of P20-Tau on MTs, which were previously assembled in the presence of P0-Tau (Fig. 6*B*, *red curve*). Adding 5 μm P20-Tau to the MT assembly reaction after reaching equilibrium caused an increase in scattering intensity but otherwise left MTs intact, as confirmed by electron microscopy (Fig. 6*B*, *red curve* and *bottom right image*). Mixing the P20-Tau with preformed MT displayed bundles of MT in electron micrographs, consistent with the higher scattering intensity (54). Performing this mixing experiment in reverse order by first incubating tubulin for 5 min with P20-Tau (no assembly) and subsequent addition of P0-Tau allowed normal assembly of MT (Fig. 6*B*, *orange curve*, also seen by microtubules in EM, *bottom middle image*). The preincubation time of tubulin alone hardly reduced its activity (Fig. 6*B*, *green curve*).

In summary, these experiments show that highly phosphorylated Tau is simply neutral with regard to MTs, and it does not interfere with the activity of unphosphorylated Tau, even if the phosphorylated Tau already contained small aggregates (*i.e.* oligomeric structures).

##### Phosphorylated Tau Oligomers Cause Spine Reduction in Primary Neurons but No Pronounced Decrease in Viability

A second hypothesis for a gain of toxic function by Tau phosphorylation is its effect on neuronal cells. This follows the widely held belief that oligomeric assemblies preceding the formation of proteinaceous aggregates are the primary pathogenic species in many protein deposition diseases (proteinopathies) (55–57). In particular, it was recently reported that hyperphosphorylated and misfolded Tau can convert into diffusible oligomers that are toxic to cells (58, 59).

To investigate the toxic properties of Tau oligomers generated from hyperphosphorylated Tau, we treated mouse primary neuronal cells with 1 μm P20-Tau for 3 h. The presence of oligomers in the aggregated mixture was confirmed by TCSPC analysis. A contribution of 25% oligomers to the average lifetime (and corresponding 75% monomers) was reached after incubating 50 μm P20-Tau in BES buffer for 3 h at 37 °C. This protein solution was then mixed into the cell's cultured (conditioned) media.

**FIGURE 7.**
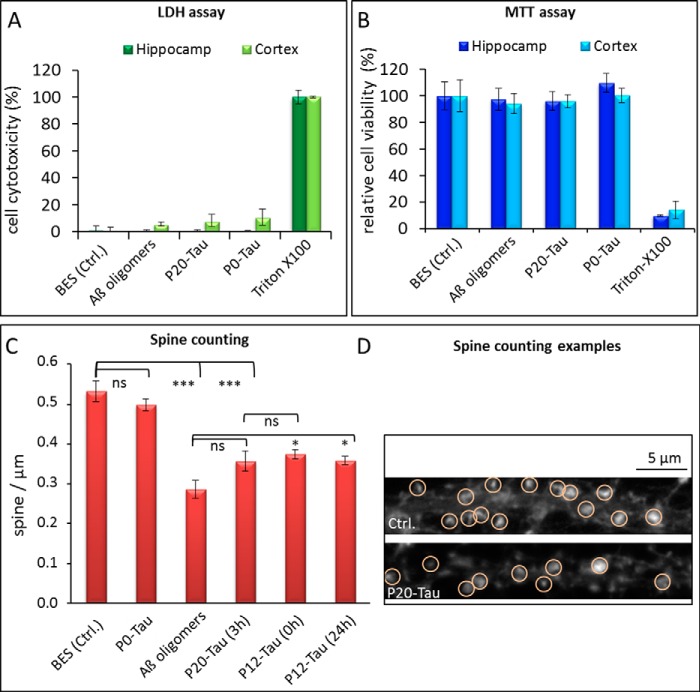
**Influence of P-Tau oligomers on the cell metabolism and dendritic spine densities in primary neuronal cells.** Cytotoxicity in primary neuronal cell culture from mouse cortex and hippocampus was tested after a 3-h incubation with 1 μm P0-Tau, P20-Tau, and Aβ-oligomers for comparison. *A,* LDH assay; *B,* MTT assays were performed from the same cultures, taking supernatant (for LDH) prior to performing the MTT assay with the remaining cells. 2% Triton X-100 was used as a toxic control (100%) and 2% buffer (50 μm BES) as a negative control. The relative cell cytotoxicity was calculated (according to the assay protocol) from the LDH release of cells after cell lysis with 2% Triton X-100 (maximal LDH release) and 2% buffer-treated cells (minimal LDH release in viable cells). Cell viability was determined by MTT assay, where the value of buffer treated cells was set to 100%. *Error bars* indicate the S.D. from *n* = 3 independent experiments. *C* and *D,* quantification of actin-enriched protrusions (spine) density on dendrites. Counting of actin-enriched protrusions (defined as dendritic spines by phalloidin stained F-actin) in primary neuronal hippocampal cultures was carried out in immunofluorescently stained cells using phalloidin as F-actin stain to visualize the protrusions (*D*). At 30 μm distance from the cell soma, a length of 20–30 μm was chosen, and the phalloidin-positive spines were counted. Primary neuronal cells were treated for 3 h with BES buffer, 1 μm Aβ oligomers (aggregated for 1 h at 37 °C (32)), and 1 μm P20-Tau (aggregated for 3 h at 37 °C). Before adding P20-Tau to the cells, part of the sample was analyzed by TCSPC (see Fig. 4). After incubating 50 μm highly phosphorylated P20-Tau for 3 h at 37 °C in BES buffer, it contained about 25% oligomers and 75% monomers. Incubating the cells for 3 h with P12-Tau, either preincubated for 0 h or 24 h at 37 °C in BES, caused also a significant spine reduction. Approximately 20 different cells were analyzed for each condition; an example of an immunofluorescence image is shown next to the diagram with examples of counted spines in *red circle. Error bars* represent STEM (*n* = 20 cells); analysis of variance test between the groups revealed *ns* = not significantly different, *p* < 0.05 (*), and highly significant for *p* < 0.001 (***) for the indicated columns.

Subsequently, we applied two toxicity assays, the LDH release assay (for cell toxicity) and the MTT assay, followed by counting spine density in primary hippocampal and cortical neuronal cultures. The LDH test allows the estimation of cell death by measuring the LDH level in the cell culture supernatant, which reflects the leakiness of membranes in degenerating cells. This analysis did not show substantially increased cell death (Fig. 7*A*). The level of released LDH was low (∼10%) compared with the reference value of 100% after destroying membranes with 2% Triton. Next, we used the MTT assay, which measures cellular metabolic activity via NAD(P)H-dependent cellular oxidoreductase enzymes and reflects the number of vital cells. Here, too, the assay results from both cortical and hippocampal neurons treated with hyperphosphorylated Tau barely diverged from the value obtained after incubation with control medium (Fig. 7*B*).

However, we found remarkable effects after exposure of neuronal hippocampal culture to 1 μm P20-Tau for 3 h on the level of dendritic spine densities. There was a 33% decrease in spine density on dendritic shafts; this compares with a 46% decrease of spines after treating the neurons with 1 μm Aβ-oligomers (Fig. 7, *C* and *D*). Analysis of variance revealed significant differences in spine density between the buffer control and P20-Tau-treated cells but no significant differences between the effects of Aβ-oligomers and highly phosphorylated P20-Tau aggregates (Fig. 7*C*). Incubating P12-Tau (freshly mixed into the media) also results in a reduced spine density (30%), as well as when the same protein was preincubated for 24 h at 37 °C in BES buffer (*F*_(5, 134)_ = 28,70; *p* < 0.001).

The results demonstrate that the treatment of primary neuronal hippocampal cells with P20-Tau-containing phospho-Tau oligomers does not change their general integrity and viability, but it has more subtle effects on the level of synaptic integrity and function, comparable with the effects of Aβ oligomers. The less phosphorylated P12-Tau is still enough to cause spine reduction, although the P20-Tau resembles the toxic effect of Aβ-oligomers more closely. The degree of aggregation by the highly phosphorylated Tau seems to be secondary.

## DISCUSSION

Because in AD phosphorylation precedes aggregation, it is often assumed that phosphorylation is the cause of aggregation. In search for a cellular model of this relationship, we noted that Tau expressed in Sf9 cells occurs in a reproducibly high state of phosphorylation, at a level that makes it comparable with AD-Tau as follows: about 12 phosphates per Tau molecule (2N4R isoform) in normal growth conditions and about 20 phosphates upon addition of the phosphatase inhibitor okadaic acid. Moreover, this high degree of phosphorylation is achieved by a variety of protein kinases active in these cells. By contrast, prokaryotic *E. coli* cells express Tau in a “naked” form without any phosphates (or other notable modifications). Remarkably, in both systems, the Tau protein is expressed at high concentrations (estimated ∼200 μm) in a fully soluble form without inclusion bodies. This is consistent with the high solubility of P0-Tau *in vitro* (∼650 μm, observed during crystallization studies). These values are much higher than the ∼1 μm estimated for neuronal cells (52, 60) and strengthen the view that even a high state of phosphorylation does not consequently lead to aggregation.

In this situation, how could one explain the pathological aggregation of one of the most soluble proteins? Several possibilities come to mind. (i) The concentration of Tau might be locally much higher than 1 μm, causing local aggregation. One contributory factor could be the macromolecular crowding in cells, which could enhance aggregation rates (61). We consider this unlikely in a healthy neuron because Tau does not aggregate in Sf9 cells despite high concentrations (see above), and because diffusion rates indicate mostly monomeric Tau in neurons (62), but traffic jams in aging neurons might give rise to varicosities with higher local levels (63). (ii) Tau might undergo (local) modifications that enhance the aggregation propensity. Proteolytic truncation of Tau is a case in point because this can generate aggregation-prone fragments (64, 65); another is the oxidation of Cys residues in the repeat domain that promotes dimerization (66). (iii) However, the most likely major factor is the interaction of Tau with cationic macromolecules that neutralize Tau's positive charges and alter its conformation. Examples are heparin or heparan sulfate proteoglycans, RNAs, arachidonic acid micelles, or acidic peptides (41, 67–69), some of which are commonly used in Tau assembly studies.

When analyzing the Sf9/BV Tau with phosphorylation-sensitive antibodies, many of the phospho-sites common with AD-Tau appeared in the repeat domain or the proline-rich flanking regions of Tau (Fig. 1*A*). The Tau from Sf9 cells also showed the upward shift of molecular weight characteristic of AD-Tau (Fig. 1, *D* and *F*), even though it remained almost fully soluble (Fig. 1*G*). Mass spectrometry by MALDI-TOF of trypsin-digested Tau Sf9/BV Tau revealed 19 phosphopeptides accounting for ∼23 P-sites, most of which were located in the basic C-terminal half of the protein, whereas the more acidic N- and C-terminal tails appeared unmodified. No modification was seen at residues Tyr-18, Tyr-197, and Tyr-394, the targets of tyrosine kinases Fyn, Abl, and others (18, 70). The only Tyr residue found in modified form was Tyr-310; this residue is part of a hexapeptide motif responsible for Tau aggregation via β-structure (71), and thus its phosphorylation may contribute to its resistance against aggregation.

Other notable sites are in the K*X*GS motifs of the repeat domain (Ser-262, Ser-324, and Ser-356), which are targets of the kinase MARK and lead to detachment of Tau from microtubules (1). The position Thr-153, a target of a proline-directed kinase, was modified as well, which may be important because the adjacent mutation A152T is a risk factor for progressive supranuclear palsy (72). For an up-to-date list of Tau phosphorylation sites observed in human brain see the Hanger lab website.

Comparison of the P12 and P20 fractions of Sf9-Tau (generated without or with okadaic acid) showed an increase in the overall extent of phosphorylation but not a pronounced change of phosphorylation sites, suggesting that a similar set of kinases is responsible. As recently shown for the case of the kinase GSK3β, a more complex picture of the phosphorylation steps is emerging when analyzed by methods that allow quantitation of phospho-sites (*e.g.* FLEXIQuant (73)). Hence, for further investigations, one should be aware of possible priming-dependent mechanisms and the resulting enhanced phosphorylation in Tau within a complex interaction of various kinases (74).

The results presented here show that even strongly hyperphosphorylated Tau had very little tendency to form insoluble aggregates. Similarly, even a combination of several well characterized phospho-residues did not cause aggregation readily (75, 76). Nevertheless, given the recent interest in the toxic functions of pretangle oligomeric Tau (77, 78), we wanted to explore the assembly behavior by several complementary spectroscopic and imaging methods, including steady-state fluorescence anisotropy, fluorescence lifetime, and electron and atomic force microscopy (Figs. 3–5). Here too, the results showed that at the initially lower concentrations of the isolated proteins (∼5 μm, Fig. 1*B, step 2*) there was no detectable assembly. However, the picture changed when the protein concentration was raised 10-fold (to 50 μm, Fig. 1*B, step 3*). In that case, the hyperphosphorylated Tau revealed an enhanced tendency for oligomerization, visualized both by the higher fluorescence anisotropy (Fig. 3, indicating slower molecular motion) and the reduced fluorescence lifetime (Fig. 4, indicating a state of higher compaction). This effect became more pronounced at longer incubation times, when the initially present oligomeric aggregation seeds (Fig. 5, *E* and *F*, *0 h*) became transformed into elongated structures. Even *bona fide* Tau fibrils with the typical PHF appearance became visible (Fig. 5, *E* and *F*; *216 h*; occasional examples even at earlier times). The oligomers and small polymers from concentrated Sf9-Tau are heterogeneous and often “amorphous,” but at least a fraction appears to be on-pathway for fibril assembly. Note that the bulk of the assembled material is still soluble, as judged by the usual Sarkosyl extraction protocol (Fig. 1*H*), and is readily dissolved under denaturing conditions, leaving only a few fibrils still detectable by EM (Fig. 2*F*).

We conclude that hyperphosphorylation enhances the tendency for oligomerization, which is not observed with nonphosphorylated protein expressed in *E. coli*. However, hyperphosphorylation alone is not sufficient for aggregation so that other promoting factors, for example an increased concentration, are necessary to induce fibril formation. In a cellular environment, this could be provided by mis-localization of Tau, which might lead to a locally increased concentration. Other post-translational modifications could have an additional impact on structural changes, as shown for the aggregation-promoting effect of Tau acetylation (79, 80).

A very different picture of the effect of phosphorylation emerges when considering the interactions of Tau with microtubules (Fig. 6). Whereas nonphosphorylated Tau readily supports MT assembly, the hyperphosphorylated Tau does not. One of the known functions of Tau is to stabilize microtubules to support growing neurites (52). The inhibition is due to the inability of phospho-Tau to interact with tubulin, because subsequent addition of nonphospho-Tau readily promotes MT assembly with normal rates. In other words, hyperphosphorylated Tau appears to be neutral with regard to MT assembly. Note that this view of “inert” phospho-Tau is different from earlier models where hyperphosphorylated Alzheimer-Tau was thought to scavenge normal Tau away from microtubules, thereby destabilizing them (53). This issue cannot be resolved at present because the distribution of P-sites and their occupancies are not known in detail, as well as other potential modifications of Tau. In our case, the simplest explanation for the apparent lack of interaction between phospho-Tau and tubulin is that this would require oppositely charged protein surfaces (cationic for Tau, anionic for tubulin). Thus, the interaction can be interrupted when a massive change of charges occurs on either partner, *e.g.* by subtilisin cleavage of tubulin (which removes the negatively charged C-terminal tail (81)) or by compensation of positive charges on Tau by phosphorylation, as described here.

Finally, one may ask what the toxic consequences of hyperphosphorylated Tau might be. For Tau variants expressed in cell models, toxic effects of large scale phosphorylation are observable but modest (26, 75), and observed changes may be better explained by specific phosphorylation sites (*e.g.* targets of GSK3β or MARK (1, 82)). Loss of microtubule stabilization by detachment of phospho-Tau would be an attractive explanation, with the caveat that the cell can compensate by up-regulation of other microtubule-associated proteins (83). When considering possible cytotoxic roles of hyperphosphorylated Tau, one should also recall that there are physiological conditions where phosphorylation of Tau is easily tolerated in cells. One example is that of fetal Tau, which displays a phosphorylation pattern similar to AD (11, 16). Moreover in hibernating animals, Tau becomes highly phosphorylated in a reversible manner, corresponding to a cyclic regression and reappearance of dendritic trees, which implies that Tau phosphorylation plays an important role for neuronal plasticity (13, 84).

Although Tau is mainly a cytosolic protein (∼1 μm in neurons (52, 60), it is also present in the brain interstitial fluid at much lower concentrations (45 ng/ml, ∼1 nm (85)). This raises the possibility that the typical progression of Tau pathology in AD might be caused by extracellular Tau, exerting toxic effects from the outside and transferring aggregation-prone Tau species to other brain areas. However, when testing extracellular Tau on primary neurons or other cell models, the effects were small, as judged by standard assays of cell viability or metabolism, even at concentrations much higher than in the interstitial fluid (Fig. 7). These match our previous observations where oligomers of the Tau repeat domain (R1 to R4) at 1 μm did not cause any viability changes in primary mouse cortex cells (86). The notable exception was the density of spines, which decreased by ∼30% when exposed to hyperphosphorylated Tau (P12 or P20) and could thus contribute to functional changes of dendritic spines, which are involved in memory impairment in AD. Apart from being in a high state of phosphorylation, these species also show an increased tendency for oligomerization (Figs. 3–5). The tendency of hyperphosphorylated Tau to keep its nonfibrillar structures matches the observed slow and progressive Tau pathology in AD with subtle changes in physiology over a long time. It is therefore possible that the toxicity lies in the oligomeric state, rather than in the phosphorylation state, which would be in line with current assumptions about the toxic functions of low-*n* Tau oligomers.

A high degree of phosphorylation of Tau is known to confer Tau resistance to proteases (87). This would slow down Tau's degradation and preserve it for rapid activation into a dephosphorylated state when necessary for MT stabilization in developing neurons or in arousal phases in hibernating animals. However, during the pathological process of AD, hyperphosphorylation of Tau resulting from inactive phosphatases, wrong compartmentalization, or additional modifications could shift the protein into the oligomeric state. This would eventually lead to the degeneration of affected neurons and finally into aggregation into neurofibrillary tangles. Further studies are required to back up these scenarios.

## Footnotes

4 The abbreviations used are:

AD: Alzheimer disease
AFM: atomic force microscopy
LDH: lactate dehydrogenase
MT: microtubules
MTT assay: (3-(4,5-dimethylthiazol-2-yl)-2,5-diphenyltetrazolium bromide
PHF: paired helical filament
TCSPC: time-correlated single photon counting
ThS: thioflavin S
BES: *N*,*N*-bis(2-hydoxyethyl)-2-aminoethanosulfonic acid
OA: okadaic acid
hTau: human Tau
Aβ: amyloid-β.
